# Establishment of a new OSCC cell line derived from OLK and identification of malignant transformation-related proteins by differential proteomics approach

**DOI:** 10.1038/srep12668

**Published:** 2015-08-03

**Authors:** Yan Dong, Qun Zhao, Xiaoyan Ma, Guowu Ma, Caiyun Liu, Zhuwen Chen, Liyuan Yu, Xuefeng Liu, Yanguang Zhang, Shujuan Shao, Jing Xiao, Jia Li, Weimin Zhang, Ming Fu, Lijia Dong, Xiandong Yang, Xu Guo, Liyan Xue, Fei Fang, Qimin Zhan, Lihua Zhang

**Affiliations:** 1 College of Stomatology, Dalian Medical University, Dalian 116044, China; 2 State Key Laboratory of Molecular Oncology, Chinese Academy of Medical Sciences and Peking Union Medical College, Beijing 100021, China; 3 Dalian Institute of Chemical Physics, Chinese Academy of Science, Dalian 116023, China; 4 Institute of Cancer Stem Cell, Second Affiliated Hospital, Cancer Center, Dalian Medical University, Dalian 116044, China; 5 Department of Engineering Mechanics, Dalian University of Technology, Dalian 116023, China; 6 Department of Pathology, Cancer Hospital and Cancer Institute, Chinese Academy of Medical Sciences and Peking Union Medical College, Beijing 100021, China

## Abstract

Oral squamous cell carcinoma (OSCC) is usually preceded by the oral premalignant lesions, mainly oral leukoplakia (OLK) after repeated insults of carcinogens, tobacco. B(a)P and DMBA are key carcinogens in tobacco smoke. In the present study, for the first time we established the cancerous cell line OSCC-BD induced by B(a)P/DMBA mixture and transformed from dysplastic oral leukoplakia cell line DOK. Cell morphology, proliferation ability, migration ability, colony formation, and tumorigenicity were studied and confirmed the malignant characteristics of OSCC-BD cells. We further identified the differential proteins between DOK and OSCC-BD cells by stable isotope dimethyl labeling based quantitative proteomic method, which showed 18 proteins up-regulated and 16 proteins down-regulated with RSD < 8%. Differential proteins are mainly related to cell cycle, cell proliferation, DNA replication, RNA splicing and apoptosis. Abberant binding function, catalysis activity and transportor activity of differential proteins might contribute to the malignant transformation of OLK. Of the 34 identified differential proteins with RSD < 8%, 13 novel cancer-related proteins were reported in the present study. This study might provide a new insight into the mechanism of OLK malignant transformation and the potent biomarkers for early diagnosis, meanwhile further facilitate the application of the quantification proteomics to carcinogenesis research.

Oral squamous cell carcinoma (OSCC) is the sixth most common cancer in the world and accounts for more than 90% of oral malignancies^1^,^2^,^3^. Carcinomas of the oral cavity, espescially OSCC, are major cause of cancer morbidity and mortality and affected nearly 500,000 patients annually world-wide^4^. Although early diagnosis and treatment are very important to the prognosis of OSCC, the specific biomarkers for early diagnosis and the valuable therapeutic targets are lacking. The prognosis is still poor with a 5-year survival rate of approximately 50%^5^.

OSCC is usually preceded by the oral premalignant lesions, mainly oral leukoplakia (OLK) after repeated insults of carcinogens, tobacco. Tobacco smoking is the most important etiological factor in the development of OLK and OSCC. Leukoplakias are oral white lesions that have not been diagnosed as any other specific disease. Gender distribution shows a strong male predominance (2:1). Prevalence of oral leukoplakia has ranged from 0.2% to 3.6%. Various studies have shown 0.6% to 20% rate of malignant transformation of OLK^6^.

Leukoplakias are white plaques in the oral mucosa and their significance lies in the fact that they have propensity for malignant transformation. OLK is the most commonly diagnosed premalignant lesion in the oral cavity and most associated with the development of OSCC. However, the mechanism of OLK malignant transformation is still not very clear. There is an urgent need to elucidate the molecular determinants and key signal pathways underlying the malignant transformation from premalignant cells to malignant cells, and to identify novel diagnostic biomarkers and therapeutic targets for OLK malignant transformation. The present study focused on the process of OLK malignant transformation and established a new OSCC cell line from OLK cells induced by tobacco carcinogens. Based on this malignant transformation cellular model, we further investigated the differentially expressed proteins between OLK cells and OSCC cells by the stable isotope dimethyl labeling based quantitative proteomics strategy to obtain the information for malignant transformation-related proteins research.

In recent years, quantitative proteomics techniques have emerged as a powerful tool to uncover the differential proteins expression associated with cancer development^7^,^8^,^9^. Chanthammachat *et al.* used two-dimensional (2D) gel electrophoresis accompanied by mass spectrometry to analyze and identify the differentially expressed proteins in 10 pairs of tumours and adjacent non-tumor tissues from five cases of early-stage and five cases of late-stage OSCC^10^. Brieger *et al.* separated and quantified the paired protein samples of 12 individuals (tongue cancer and non-cancerous mucosa) by 2D gel electrophoresis followed by MALDI-TOF mass spectrometry identification to explore the differentially expressed proteins for potential biomarkers and therapeutic targets of OSCC^11^. However, the drawbacks of 2D gel electrophoresis, such as low sensitivity, low-resolution and high loss triggered the development of shotgun based stable isotope labeling quantitative proteomic strategies. Nowadays, stable isotope dimethyl labeling based quantitative proteomic method is one of the most popular techniques for quantitative proteomic analysis with the advantages of universality, rapid and high derivatization efficiency^12^. Furthermore, *in vitro* cellular model can be used as a simplified model system for studying changes that accompany malignant transformation. It is an indispensable study tool in researching for molecular mechanism because of homogeneity of cell population, accessibility, reproducibility and hence enough amount of material for analysis^13^.

Some studies established OSCC cell lines from human oral epithelial cells transfected with HPV16 E6/E7 genes^14^,^15^,^16^,^17^. Transfection with viral genes could induce chromosomal rearrangements or abnormal genes expression. Furthermore, leukoplakia is a clinical term which is based on the exclusion criterion after excluding other white lesions such as lichen planus, leukoedema, etc. OLK cell line derived from OLK clinical tissues is more suitable to study the mechanism of OLK malignant transformation than oral epithelial cell lines. Therefore, for the first time the present study established the cancerous line OSCC-BD from dysplastic oral leukoplakia cell line DOK (Dysplastic Oral Keratinocyte) by induction with tobacco carcinogens. DOK cell line is from a piece of dorsal tongue showing epithelial dysplasia. The tissue was obtained from a 57-year-old man who was a heavy smoker prior to the appearance of a white patch on his tongue. Subsequently a squamous cell carcinoma developed at the site^18^.

Tobacco contains many carcinogens. Benzo[a]pyrene [B(a)P] and 7,12-Dimethylbenz(a)anthracene (DMBA) are important carcinogens in cigarette smoke. B(a)P is a polycyclic aromatic hydrocarbon and a potent inducer of carcinogenesis. Previous reports showed that bioactivation of B(a)P binds to the cellular DNA in human cells and the DNA adducts formed have been postulated to be central to the carcinogenic process^19^. Previous studies established OSCC cell lines induced by B(a)P from human oral epithelial cells transfected with HPV16 E6/E7 genes and further identified CK17 expression upregulated in OSCC cells by using two-dimensional (2D) gel electrophoresis and liquid chromatography-tandem mass chromatography^7^,^16^. DMBA, a potent organ and site specific carcinogen, is commonly used to induce buccal pouch carcinogenesis in hamsters. Some researches demonstrated DMBA-induced DNA damage and mutations in experimental animal models^20^. These studies mentioned above established the carcinogenesis cell or animal models by induction with B(a)P or DMBA alone. As we known, tobacco smoke is a mixture of many carcinogens. In the present study, the cigarette smoke are simulated using a mixture of B(a)P and DMBA. OSCC cell line induced by B(a)P/DMBA mixture might be better than by B(a)P or DMBA alone to study the mechanism of OLK malignant transformation. So we established cancer cell line OSCC-BD induced by B(a)P/DMBA mixture from DOK cells.

On the basis of this new malignant transformation cellular model, we investigated the differentially expressed proteins between DOK cells and OSCC-BD cells by the stable isotope dimethyl labeling based quantitative proteomics strategy to provide new clues to potent biomarkers and therapeutic targets for OLK malignant transformation.

## Results

### Establishment of the cell line OSCC-BD

Human dysplastic oral leukoplakia cell line DOK were cultured with DMEM and treated intermittently with 70 μM B(a)P/DMBA mixture for three months. To confirm the malignant characteristics of the transformed cell line OSCC-BD, cell morphology, proliferation ability, migration ability, colony formation in soft agar, and tumorigenicity were studied.

### Morphology Change of OSCC-BD cells

**Figure 1:**
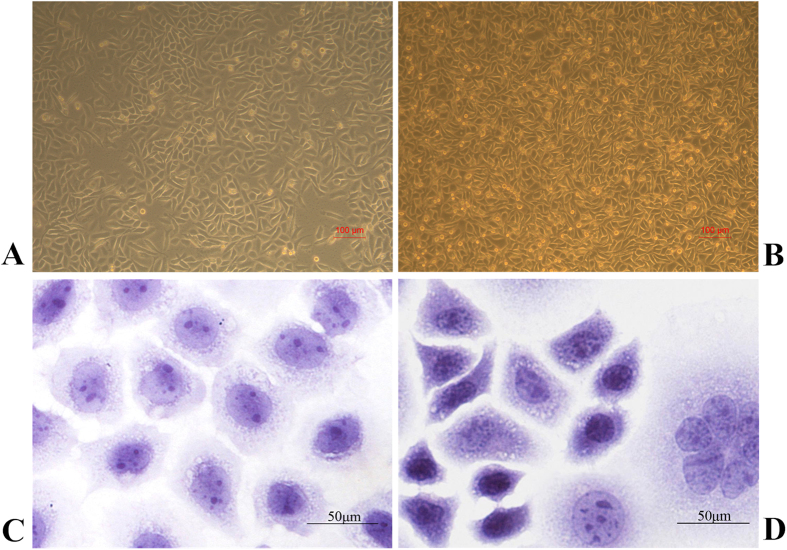
Different morphology between DOK cells and OSCC-BD cells. (**A**) DOK cells showed obvious contact inhibition and (**B**) OSCC-BD cells overlapped with loss of contact inhibition. (**C**) HE staining showed DOK cells were cobble stone-like in shape and remained almost uniform in size. (**D**) OSCC-BD cells showed malignant characters with increased ratio of nucleus and plasma, more cell division, and sporadic tumor giant cells.

After DOK cells were treated with B(a)P/DMBA mixture for 3 months, OSCC-BD cells had been cultured *in vitro* for more than 20 months and over 100 passages in DMEM. Using the inverted contrasting microscope (Leica DMIL, Germany), DOK cells showed obvious contact inhibition (Fig. 1A) and OSCC-BD cells showed uncontrolled cell division with overlapping (Fig. 1B). DOK cells showed the character of epithelial dysplasia, cobble stone-like in shape, and remains almost uniform in size by HE staining (Fig. 1C). OSCC-BD cells also showed the character of epithelial, however, the increased ratio of nucleus and plasma, more cell division, and sporadic tumor giant cells showed the malignant character (Fig. 1D).

### Comparative analysis of cell growth between DOK and OSCC-BD cells

**Figure 2:**
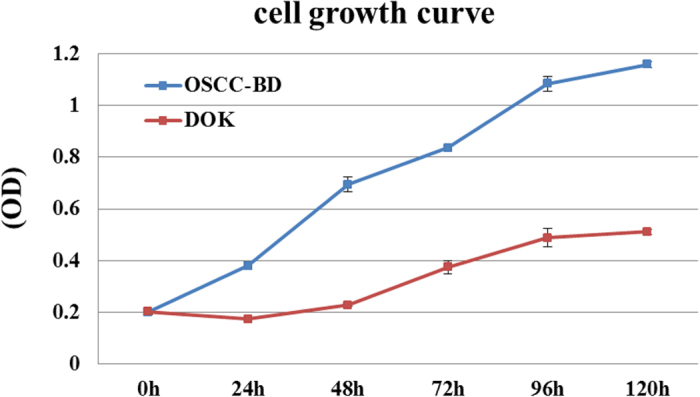
Comparative analysis of proliferation ability in DOK and OSCC-BD cells. DOK and OSCC-BD cells were subcultured for 1, 2, 3, 4 and 5 days. The cell growth curves showed OSCC-BD cells grew significantly faster than DOK cells and an approximately 2-3-fold increase from day 1 to day 5 in OSCC-BD cells compared to DOK cells was observed (*p* < 0.05).

DOK and OSCC-BD cells were seeded and were subcultured for 1, 2, 3, 4 and 5 days, respectively. We compared the optical density at 492 nm to assess the degree of cell survival. From the cell growth curve by MTS assay (Fig. 2), OSCC-BD cells grew significantly faster than DOK cells (*p* < 0.05). Cell proliferation in OSCC-BD cells showed an approximately 2-3-fold increase from day 1 to day 5, compared to DOK cells.

### Increased percentage of OSCC-BD cells in S phase

**Figure 3:**
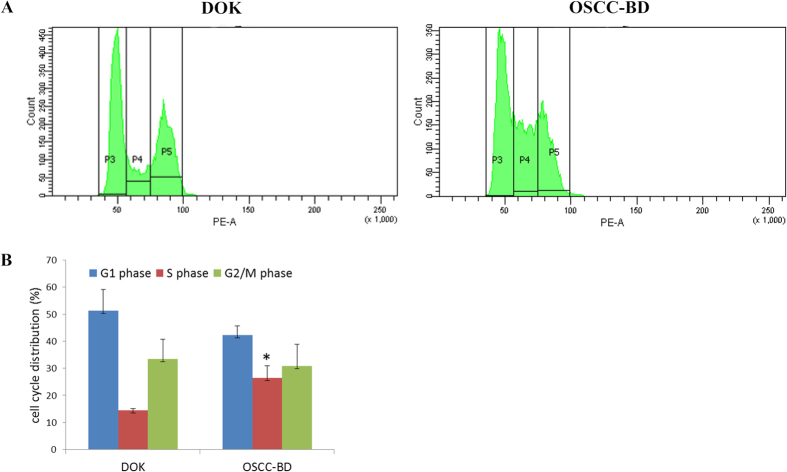
The cell cycle distribution of DOK cells and OSCC-BD cells by flow cytometry. (**A**) Flow cytometric results showed an accumulation of OSCC-BD cells in S cell-cycle phase. (**B**) The histogram showed the percentage of OSCC-BD cells in S phase (26.4 ± 4.6%) was significantly higher than that of DOK cells (14.5 ± 0.6%). Data are expressed as mean ± SD of three independent experiments done. *p < 0.05.

DOK cells and OSCC-BD cells were subcultured for 48 h and harvested, then were stained with propidium iodide and measured using a flow cytometer. Flow cytometric results presented cell cycle distribution (Fig. 3A). B(a)P/DMBA mixture significantly induced an accumulation of OSCC-BD cells in S cell-cycle phase. The percentage of OSCC-BD cells in S phase (26.4 ± 4.6%) was significantly higher than that of DOK cells (14.5 ± 0.6%) (*p* < 0.05, Fig. 3B). No obvious change was found between OSCC-BD and DOK cells in G1 and G2/M phases (*p* > 0.05).

### Increased colony forming capacity in OSCC-BD cells

**Figure 4:**
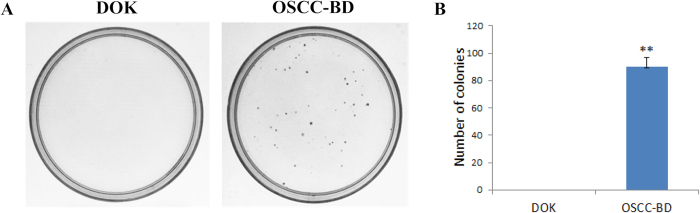
Colony formation assay of DOK and OSCC-BD cells. (**A**) In soft-agar colony formation test, 500 cells from each type of cell line were incubated for 14 days respectively. Only colonies with diameter > 100 μm were counted. OSCC-BD cells formed many colonies in soft agar and no colony formation was observed in DOK cells. (**B**) Graphical representation of colony forming assay showed OSCC-BD cells formed 90 ± 7 colonies and DOK cells did not form colonies. The colony-forming ability of OSCC-BD cells has been dramatically increased compared with DOK cells. Results from three independent experiments were quantified. **p < 0.001.

The ability to form colonies in soft agar was assessed in OSCC-BD and DOK cells. OSCC-BD cells and DOK cells were allowed to grow in 0.35% agarose in DMEM supplemented with 20% FBS for 14 days, respectively. Results from three independent experiments were quantified. The number of colonies formed in OSCC-BD cells increased compared with DOK cells (Fig. 4A). As we can see in Fig. 4B, OSCC-BD cells formed 90 ± 7 colonies/well in soft agar and DOK cells did not form colonies. The ability to form colonies in OSCC-BD cells was significantly higher than that in DOK cells (*p* < 0.001).

### Increased tumorigenicity of OSCC-BD cells in nude mice

**Figure 5:**
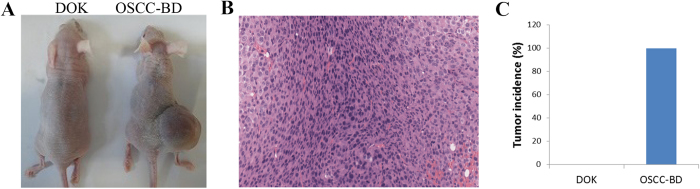
Comparison of tumorigenicity between DOK and OSCC-BD cells. (**A**) Two groups of 8 nude mice were rejected with DOK and OSCC-BD cells, respectively. Tumor formation was observed in nude mice inoculated with OSCC-BD cells and neoplasms were about 1.0 ± 0.2cm in size. No tumor was observed in those mice inoculated with DOK cells. (**B**) HE staining showed the neoplasm was typical squamous cell carcinoma. (**C**) The graphy represented the tumor formation rate of OSCC-BD cells was 100% and DOK cells didn’t form tumors.

Two groups of nude mice (8 each) were used to analyze *in vivo* tumorigenicity of DOK and OSCC-BD cells, respectively. Following inoculation of 5 × 10^6^ cells/mouse, tumors were observed in 8 of 8 (100%) tested nude mice inoculated with OSCC-BD cells. Figure 5A shows the representative mice with tumor mass which were inoculated with OSCC-BD cells, and no tumor was observed in those mice inoculated with DOK cells. HE staining showed that tumors formed were typical squamous cell carcinoma (Fig. 5B). In the nude mice injected with OSCC-BD cells, hard neoplasm formed was about 1.0 ± 0.2 cm in size and the rate of neoplasm formation was 100% (Fig. 5C).

### Increased migration ability *in vitro* in OSCC-BD cells

**Figure 6:**
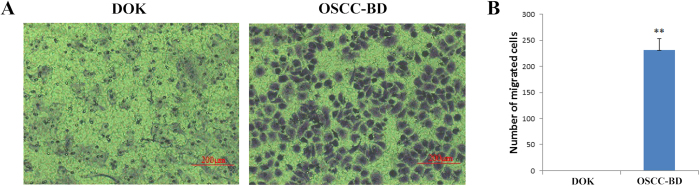
DOK and OSCC-BD cell migration assay using a transwell cell culture system. (**A**) A great number of OSCC-BD cells migrated and DOK cell migration was not observed. (**B**) The number of migrated OSCC-BD cells was 232.7 ± 20.9 per visual field with a microscope at 100 × magnification and no DOK cells migrated. Migrated cell number is shown from the results of three independent experiments. **p < 0.001.

Migration ability of OSCC-BD cells was assessed using transwell migration assays. OSCC-BD and DOK cells grew well in the transwell chamber. After the cells were stained with Giemsa, the migrated cells to the lower surface of the insert was determined. Figure 6A showed a great number of OSCC-BD cells migrated and no DOK cells migration was observed. The number of the migrated OSCC-BD cells was 232.7 ± 20.9 per visual field with a microscope at 100 × magnification. This indicates that migration ability of OSCC-BD cells was significantly stronger than that of DOK cells (*p* < 0.001, Fig. 6B).

### Decreased expression of p53 and Rb proteins in OSCC-BD cells

**Figure 7:**
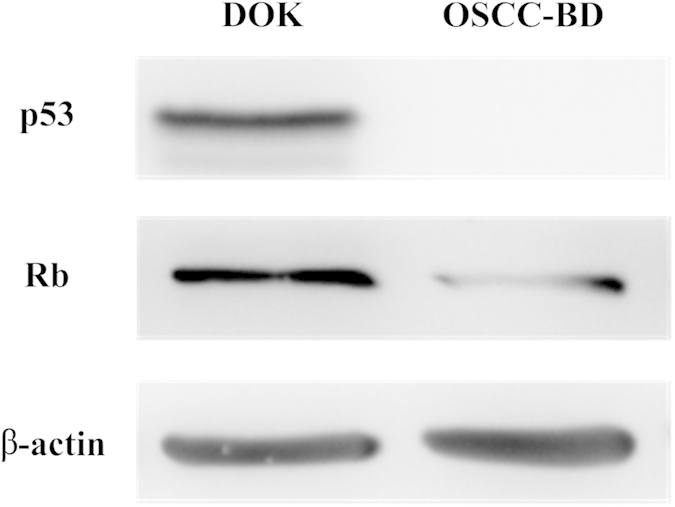
Tumor suppressor p53 and Rb proteins expression in DOK and OSCC-BD cells. Expression of p53 and Rb proteins by Western blotting assay was significantly lower in OSCC-BD cells than in DOK cells. p53 protein expression in OSCC-BD cells could not be observed.

Expression of tumor suppressor p53 and Rb proteins was evaluated by Western blotting assay. The results showed significantly lower expression of p53 and Rb proteins was found in OSCC-BD cells than in DOK cells and p53 expression did not be observed in OSCC-BD cells (Fig. 7).

### Differential proteomic analysis between DOK and OSCC-BD cells

Stable isotope dimethyl labeling based quantitative proteomics strategy was applied for differential proteins discovery between DOK and OSCC-BD cells. To verify the accuracy of quantitative method for oral squamous cell carcinoma cell line, 6 × 10^5^ OSCC-BD cells were equally split into two parts and labeled with regular and deuterated formaldehyde, mixed together and analyzed triply by nanoLC-ESI-MS/MS.

279 proteins were quantified and the log2 ratios of all the proteins fell in the range of [-1, 1] (Supplementary Figure S1), within which no significant variation in proteome quantification is usually considered. In addition, the CVs of the ratios for these 279 proteins with replicate quantification were 8.06%, demonstrating the highly accurate and precise quantification results could be achieved by our method. Based on this, we further applied this quantification strategy for DOK and OSCC protein sample differential analysis. As a result, 534 proteins were quantified at least twice when controlling the RSD of the ratios for the quantified proteins to less than 50%, of which, 154 proteins were quantified with at least a 2-fold change (Supplementary Excel File).

To characterize 154 protein groups quantified, the distributions of cellular components and molecular functions of identified proteins were further analyzed, according to Gene Ontology (GO) information obtained with GoMiner. 154 and 150 proteins were of annotated cellular component and molecular function, respectively. Most of identified annotated proteins were mapped on the organelles of nucleus (25%) and macromolecular complex (25%). Besides, 15% and 14% of identified proteins were located in mitochondrion and plasma membrane, respectively. Other proteins were from cytoplasmic membrane-bounded vesicle, endoplasmic reticulum, Golgi apparatus, ribosome and centrosome (Supplementary Figure S2A). In addition, GO molecular function annotation analysis revealed that 43% of the identified annotated proteins were of binding function, followed by catalytic activity of 24% and transporter activity of 7%, as shown in Supplementary Figure S2B.

Furthermore, 34 proteins with RSD < 8% were confidently quantified, of which 18 proteins exhibit up-regulated (ratio > 2) and 16 proteins exhibit down-regulated (ratio < 0.5) in the replicated analyses. Among these 34 differential proteins, 13 proteins were reported in this study for the first time, whose functions in human tumorigenesis are unknown. The detailed information about 34 differential proteins quantified is listed in Supplementary Table S1,S2,S3. Table S1 is for upregulated proteins, Table S2 is for downregulated proteins and Table S3 is for novel cancer-related proteins. According to the NCBI database (http://www.ncbi.nlm.nih.gov) description, we found most proteins are related to cell cycle, cell proliferation, DNA replication, RNA splicing, apoptosis and adhension. To explore the associations among differentially expressed proteins, we used the IPA software to visualize the potential biological pathways (Supplementary Table S1,S2,S3). The results showed some signaling pathways, such as signaling by Rho family GTPases, RhoA signaling, mitochondril dysfunction and 14-3-3-mediated signaling might be closely related to OLK malignant transformation.

## Discussion

The mechanism of malignant transformation from premalignant lesions to malignant tumors is very complex and remains poorly understood. In the present study, under the induction with B(a)P/DMBA mixture, DOK cells are transformed to cancer cells OSCC-BD. OSCC-BD cells showed the uncontrolled cell division with overlapping. By HE staining OSCC-BD cells showed the malignant character, the increased ratio of nucleus and plasma, more cell division, and sporadic tumor giant cells. From the cell growth curve by MTS assay, cell proliferation in OSCC-BD cells showed an approximately 2-3-fold increase, compared to DOK cells (*p* < 0.05). The flow cytometric assay further confirmed the increased proliferation ability in OSCC-BD cells, consistent with the MTS results. OSCC-BD cells showed a significant accumulation in S phase (*p* < 0.05). Colony formation assay revealed a high ability to form colonies in the OSCC-BD cells. In the nude mice experiments the rate of neoplasm formation of OSCC-BD cells was 100%. Transwell migration assays suggested a strong migration ability in OSCC-BD cells. Significantly decreased expression of tumor suppressor p53 and Rb proteins was found in OSCC-BD cells.

Taken together, all these tests confirmed the malignant characteristics of OSCC-BD cells. This new cellular model could be used as an important and ideal study tool to investigate the mechanism of OLK malignant transformation. By now a few studies were done on differential analysis of proteomic profiles between premalignant lesions and invasive cancers. Based on our malignant transformation cellular model, we performed a comparative proteomic analysis to profile differentially expressed proteins in the malignant transformation process.

We compared the quantified differential proteins in our dataset with the results obtained using clinical tissues. As a result, some of the significant proteins are both identified in tissue and cell samples, which involved in cellular processes essential for cell growth, survival and cell migration, and so on forth. For example, protein 14-3-3 s epsilon as a member of S100A7, is an intracellular binding protein which regulates different signaling processes related to carcinogenesis. In our results, the overexpression of 14-3-3 σ has been found in OSCC-BD cells, in accordance with the results previously reported in oral tumor compared to non-tumorous mucosa^21^. As another example, HSP family members are related to cancer cell growth through an anti-apoptotic property^10^. In our results, Isoform 2 of heat shock protein HSP 90-alpha and isoform alpha of heat shock protein 105 kDa were also up-regulated in OSCC-BD cells, which have been reported in many studies of OSCC tissue samples^22^,^23^. Besides, many other differential proteins, such as Endoplasmin, Succinyl-CoA:3-ketoacid-coenzyme A transferase 1, mitochondrial, and so on, were exclusively identified with our method, which might attribute to the reasons of the advisable cell model establishment, the efficient sample preparation as well as the improved nanoLC-ESI-MS/MS analysis with long separation column and high resolution mass spectrometer.

As shown in Supplementary Figure 2A and 2B, we also analyzed the cellular location and biological process involved. The results suggested that malignant transformation of OLK might mainly locate on the organelles of nucleus, macromolecular complex, mitochondrion and plasma membrane. Molecular function analysis suggested that abberant binding, catalysis and transport process might be closely related to OLK malignant transformation.

We further analyzed 34 quantified differential proteins with RSD < 8% listed in Supplementary Table S1,S2,S3 and the results suggested that minichromosome maintenance proteins (MCMs) might play an important role in malignant transformation process. MCMs are a group of proteins closely related to the DNA replication, consisting of MCM2(BM28), MCM3(HCC5), MCM4(Cdc21), MCM5(Cdc46), MCM6(Mis5) and MCM7(Cdc47). MCM proteins are essential for the initiation of eukaryotic genome replication. They are important members of replication licensing factor(RLF) which indicate the start of the initiation of DNA replication bonding with the chromation before the initiation of DNA replication. They are only present in the nucleus throughout the cell cycle. MCM proteins are proliferative markers that have been proposed as diagnostic markers in many cancers. MCM4 protein encoded by MCM4 gene is one of the highly conserved MCM proteins. In the present study, MCM4 expression showed an approximately 4-fold increase in OSCC-BD cells compared with DOK cells, consistent with the previous results showing MCM4 overexpression in human cancers, such as esophageal cancer, cervical squamous cell carcinoma and gastric cancers^24^,^25^,^26^. BM28, also known as MCM2, is also one of MCM proteins, which play a critical role in DNA replication by helping to ensure that DNA is replicated once and only once per cell cycle. In the present study BM28 overexpression was identified in OSCC-BD cells, consistent with the previous reports showing a significantly stronger immunoblot signal of BM28 in human tumors than in normal tissues^27^.

As shown in Supplementary Table S1 and S2, EZR and SEPT9 proteins expression was 3-4 fold higher in OSCC-BD cells than in DOK cells. VIM proteins expression showed 4 fold decrease in OSCC-BD cells compared to DOK cells. Using the IPA software, EZR, SEPT9 and VIM proteins were found involved in signaling by Rho family GTPases, which suggested that signaling by Rho family GTPases might be closely related to the malignant transformation of OLK. EZR, SEPT9 and VIM proteins might play key roles in the process of malignant transformation by Rho family GTPases pathway. The Rho family of GTPases is a family of small signaling G proteins. The members of the Rho GTPase family have been shown to regulate a broad diversity of cellular functions including cytoskeletal organization, membrane trafficking, cytokinesis, cell proliferation, cell motility and transcriptional regulation. Septins are a large family of GTP-binding proteins abnormally expressed in many solid tumors. Septin 9 (SEPT9) is involved in cytokinesis and cell cycle control. It has been found overexpressed in diverse human tumors including breast, head and neck, ovarian, endometrial, kidney, and pancreatic cancer^28^,^29^, consistent with the present results obtained in OSCC-BD cells. The results suggested the significance of SEPT9 as a promising tool in cancer detection. Ezrin (EZR) is known to be involved in intercellular interactions, and a shift from membrane-bound to cytoplasmatic protein expression has been associated with malignant potential. Some reports showed that Ezrin overexpression was involved in invasion, metastasis, and poor prognosis in various cancers including breast cancers, esophageal squamous cell carcinoma (ESCC) and uterine cervical cancer^30^,^31^,^32^. Our results also showed upregulated Ezrin expression in OSCC-BD cells. Vimentin(VIM) protein is a member of the intermediate filament family and responsible for maintaining cell shape, integrity of the cytoplasm, and stabilizing cytoskeletal interactions. Korsching E *et al.* reported that Vimentin expression is rather rare in breast cancer and 7.7% of 364 breast cancer tissue samples expressed vimentin by immunohistochemistry^33^. In the present study decreased expression of VIM was also observed in OSCC-BD cells compared to DOK cells. The results suggested that decreased VIM expression might be involved in the malignant transformation process.

To further analyze the 34 quantified differential proteins with RSD < 8%, we referred to the previous reports and found out that most of the 34 quantified proteins expression were consistent with the results obtained in previous studies of human cancers. Among 34 differential proteins, 18 proteins exhibited up-regulated (ratio > 2) and 16 proteins exhibit down-regulated in OSCC-BD cells (ratio < 0.5). Our most significant finding was that 13 novel cancer-related proteins, including 5 proteins upregulated and 8 proteins downregulated in OSCC-BD cells were identified to explore the early biomarkers for OLK malignant transformation.

The molecular mechanisms of the 13 novel cancer-related proteins in the tumorigenesis are still unknown and should be further elucidated. According to the NCBI database description, 13 novel cancer-related proteins may be closely related to the tumorigenesis. IKBIP protein is related to apoptosis, SRSF7 and TARDBP proteins are related to RNA splicing, OXCT and ERP70 proteins are related to metabolic process, EDMD protein is related to regulation of Wnt signaling pathway, NDUFV2 protein might be involved in mitochondril dysfunction, H2AFC protein is core component of nucleosome and related to nucleosome assembly, NFU1 protein is related to the iron-sulfur cluster assembly; According to GO molecular function annotation analysis, EDMD, CLTB, SRSF7, NHP2L1 and TMED10 were of binding function, CLTB, RAB11FIP1 and TMED10 were of protein transporter activity. The function studies for these 13 novel cancer-related proteins were still under way in our lab.

Taken together, our work lays the foundation for novel exploration of OLK malignant transformation and further facilitate the application of the quantification proteomics to carcinogenesis research.

## Methods

### Cell culture and induction with B(a)P/DMBA mixture

DOK cells were cultured in Dulbecco’s modified Eagel’s medium (DMEM), 20% fetal bovine serum (FBS) and 5 μg/ml hydrocortisone. DOK cells were treated intermittently and gradually with 70 μM B(a)P/DMBA mixture for three months. The transformed cells were named as OSCC-BD cells.

### Assay of cell growth

To validate the proliferation ability of OSCC-BD cells, the assay of cell growth was performed. DOK and OSCC-BD cells were seeded respectively in 96-well plate with 2.0 × 10^3^ cells per well. The cells were subcultured at 37°C in a 5% CO_2_ incubator for 1, 2, 3, 4 and 5 days, respectively. 20 μl of 3-(4,5-dimethylthiazol-2-yl)-5-(3-carboxymethoxyphenyl)-2-(4-sulfophenyl)-2 H-tetrazolium solution (MTS) (Promega) was added to each well and then incubated for a further 4 h. Optical density (OD) was then read directly at 492 nm using the iMark Microplate Reader (Bio-Rad, USA). The plates were also examined under the microscope to assess the degree of cell survival.

### Cell cycle analysis

In order to confirm the MTS results, the cell cycle of DOK cells and OSCC-BD cells was analyzed using a flow cytometer. DOK cells and OSCC-BD cells were subcultured for 48 h, and dispersed by trypsinization and suspended in PBS, then the cells were stained with propidium iodide and measured using a flow cytometer (LSR II, BD Biosciences, USA). The cell cycle distribution was analyzed by computer software (ModFit3.2, Verity Software House, USA). The procedure was performed according to the manufacturer’s protocol.

### Soft agar colony formation assay

To explore the colony formation ability of DOK and OSCC-BD cell lines, soft agar colony formation assay was done. Agar plates were prepared by first applying a base layer of 0.6% agar in DMEM. Over this basal layer, an additional layer of 0.35% agar and 500 cells/well were added. DOK cells and OSCC-BD cells added were incubated at 37 °C in a 5% CO_2_ incubator for 14 days respectively. Only colonies with diameter > 100 μm were considered. The results were expressed as the number of colonies formed in each type of cell.

### Tumorigenicity

The *in vivo* tumor formation assay was performed by injecting subcutaneously DOK cells and OSCC-BD cells respectively into SPF BALB/c nude mice (aged 4 weeks, and weighted 18–22 g) with 5 × 10^6^ cells suspended in 200 μl of sterile phosphate-buffered saline per mouse. Two groups of nude mice (8 each) were used to analyze tumorigenicity of DOK and OSCC-BD cells, respectively. After one month, the mice were killed. The tumors were fixed in 10% neutralized formalin, and embedded in paraffin. Each sample was studied using the hematoxylin and eosin (HE) staining. The animal experiments were carried out in accordance with the approved guidelines and all experimental protocols were approved by the Animal Care and Use Committee of Cancer Hospital and Cancer Institute in Chinese Academy of Medical Sciences and Peking Union Medical College.

### *In vitro* migration ability

To investigate the migration ability of OSCC-BD cells, a transwell cell culture system was used. The upper surface of transwell was coated with 2% Matrigel (BD Biosciences, USA). DOK cells and OSCC-BD cells at a density of 1.8 × 10^5^ were seeded in the upper chamber of each well with 100 μl serum-free DMEM. 600 μl 20% FBS-DMEM was placed in the lower chambers. The cells were allowed to migrate at 37 °C in a 5% CO_2_ incubator for 12 h. Then the cells were fixed in methanol and stained with Giemsa. Cell migration was determined by the number of the cells that had migrated to the lower surface of the insert with a microscope at 100 × magnification.

### Western blot analysis

To explore expression of p53 and Rb proteins in DOK cells and OSCC-BD cells, Western blotting was performed. The procedure was performed as previously used^34^. β-actin was used as internal control protein. The following antibodies were used: anti-p53 antibody (dilution 1:1000) (Santa Cruz Biotechnology, USA), anti-Rb antibody (dilution 1:500) (Santa Cruz Biotechnology, USA), and anti-β-actin antibody (dilution 1:5000) (Sigma, USA). Three independent experiments were done.

### Protein extraction and dimethyl labeling

Based on the cell model of carcinogenesis established in the present study, differential proteomics analysis of DOK cells and OSCC-BD cells was carried out. Six batches of cells were merged to collect and homogenized in 1 ml lysis buffer (8 M urea, 1 × PBS and 1% (v/v) protease inhibitor cocktail) using ultrasonication (Cole-Parmer, Vernon Hills, IL, USA) for 5 min on ice at 100% pulse power to break the cells and extract proteins. Protein lysates were centrifuged for 40 min at 2 × 10^4^ rpm, supernatants were collected, and protein concentrations were determined by Bradford assay (BioRad, Munich, Germany).

Then, equal aliquots of DOK and OSCC cell lysate were reduced with DTT (final concentration, 10 mM), and subsequently alkylated with iodoacetic acid (final concentration, 25 mM), respectively. Each of the resultant protein solution was diluted to 1 M urea in 50 mM NH_4_HCO_3_ (pH 8.0), and then incubated with trypsin (Promega, Madison, WI, USA) at 37 °C for 16 h. The solution was desalting using C18 trap column. The collected peptides were dried with a SpeedVac and reconstituted in 50 mM phosphate buffer (pH 7.2). After that, the tryptic peptides of DOK cells and OSCC cells were labeled with isotopomeric dimethyl labels.

Briefly, for every 100 μg of tryptic peptides, 4 μl of 4% (v/v) formaldehyde (CH_2_O) and 4 μl of 0.6 M cyanoborohydride (NaBH_3_CN) were added for the dimethylation reaction of peptides from DOK cells, while 4 μl of 4% (v/v) formaldehyde (CD_2_O) and 4 μl of 0.6 M cyanoborohydride (NaBH_3_CN) were added for the dimethylation reaction of peptides from OSCC cells. After keeping the reaction solutionin 37 °C for 1 h, 2 μl of 10% (v/v) ammonia and 5 μl of 10%(v/v) formic acid in water were successively added to quench the reaction. Finally, the labeled peptides of DOK and OSCC cells were pooled and stored at −80 °C until LC-MS/MS analysis. To validate the performance of dimethyl labeling strategy as well as sample preparation for DOK and OSCC cell sample analysis, two identical tryptic peptides of OSCC sample (each from 3 × 10^5^) were differentially labeled and equivalently mixed for RP-LC-ESI- MS/MS analysis.

### NanoL-CESI-MS/MS Analysis

The peptide samples were analyzed by nano-RPLC-ESI-MS/MS with an LTQ-OrbitrapElite mass spectrometer equipped with a Dionex ultimate 3000 liquid chromatography and an ESI probe Ion Max Source with a nanospray kit. The spectrometer was controlled by Xcalibur software version 2.2 (Thermo Fisher, Waltham, MA, USA). The peptides were separated on a C18 capillary column (30 cm, 75 μm i.d./375 μm o.d.) packed with C18 silica particles (5 μm, 100 Å) with a 160 min gradient from 10 to 35% acetonitrile and analyzed on the mass spectrometer. Mass spectra were acquired in a data-dependent mode. MS1 spectra were measured at a resolution of 6 × 10^4^ and the top 15 most abundant ions with an isolation window of 2 m/z were selected for sequencing and fragmented in the data-dependent CID mode with a normalized collision energy of 35%, activation Q of 0.25, activation time of 10 ms, and one microscan. The sample was analyzed in triplicate.

### Statistical analysis

All data from tests for cell model validation were analyzed using the statistical software package SPSS for Windows version 10.0 (SPSS Inc., USA). The results were expressed as means ± S.D. for at least triplicate determinations and T-test was performed. When the *p*-value was less than 0.05, the difference was regarded as statistically significant. All raw files from nanoLC−ESI-MS/MS analysis were processed in MaxQuant, version 1.2.2.5 and the Andromeda search engine against the IPI human database (version 3.87, 91 464 entries). Peptides were searched using the following parameters: fully tryptic cleavage constraints; up to two internal cleavage sites allowed for tryptic digestion; carbamidomethylation as a fixed modification; oxidation of methionine and protein N-terminal acetylationas variable modifications; dimethyl (+28.0313 Da) and dimethyl (+32.0564) N-termini and K set as light/heavy labels for quantification; a 20-ppm first-search tolerance and a 6-ppm main-search tolerance; minimum peptide length was set to six residues; the false discovery rate for all peptides, PTM sites, and protein identifications was set to 0.01. Proteins quantified at least twice and with at least a 2-fold change were considered to be differentially expressed proteins^35^. The cellular components and molecular functions based on Gene Ontology (GO) consortium were assigned with GoMiner. The molecular functions and biological process of the differential proteins were annotated according to the Uniprot database (http://www.uniprot.org/). The differential proteins were mapped to existing pathways using IPA software to visualize the potential cancer-related pathways.

## Additional Information

**How to cite this article**: Dong, Y. *et al.* Establishment of a new OSCC cell line derived from OLK and identification of malignant transformation-related proteins by differential proteomics approach. *Sci. Rep.* **5**, 12668; doi: 10.1038/srep12668 (2015).

## Supplementary Material

Supplementary Excel File

Supplementary Information
