# Supplementary material for: Establishment of a new OSCC cell line derived from OLK and identification of malignant transformation-related proteins by differential proteomics approach
Source: Sci Rep. 2015 Aug 3;5:12668. doi: 10.1038/srep12668 (PMC4522661; doi:10.1038/srep12668)
Supplement: Supplementary Information [file srep12668-s2.pdf]

Establishment of a new OSCC cell line derived from OLK and identification of malignant transformation-related proteins by differential proteomics approach

Yan Dong[1,2]*, Qun Zhao[3], Xiaoyan Ma[2,1], Guowu Ma[1], Caiyun Liu[2,1], Zhuwen Chen[1], Liyuan Yu[2,1], Xuefeng Liu[4], Yanguang Zhang[2,1], Shujuan Shao[4], Jing Xiao[1], Jia Li[2], Weimin Zhang[2], Ming Fu[2], Lijia Dong[2], Xiandong Yang[1], Xu Guo[5], Liyan Xue[6], Fei Fang[3], Qimin Zhan[2]*, Lihua Zhang[3]*

The first two authors are co-first author.

[1]College of Stomatology, Dalian Medical University, Dalian 116044, China; [2]State Key Laboratory of Molecular Oncology, Chinese Academy of Medical Sciences and Peking Union Medical College, Beijing 100021, China; [3]Dalian Institute of Chemical Physics, Chinese Academy of Science, Dalian 116023, China; [4]Institute of Cancer Stem Cell, Second Affiliated Hospital, Cancer Center, Dalian Medical University, Dalian 116044, China; [5]Department of Engineering Mechanics, Dalian University of Technology, Dalian 116023, China; [6]Department of Pathology, Cancer Hospital and Cancer Institute, Chinese Academy of Medical Sciences and Peking Union Medical College, Beijing 100021, China.

Corresponding Authors: Yan Dong, College of Stomatology, Dalian Medical University, Dalian 116044, China; State Key Laboratory of Molecular Oncology, Cancer Hospital and Cancer Institute, Chinese Academy of Medical Sciences and Peking Union Medical College, Beijing 100021, China. Phone: 86-411-86110401, Fax: 86-10-67715058, E-mail: dongyanzy2011@163.com; Lihua Zhang, Dalian Institute of Chemical Physics, Chinese Academy of Science, Dalian 116023, China. Fax: 86-411-84379560, E-mail: LihuaZhang@dicp.ac.cn; Qimin Zhan, State Key Laboratory of Molecular Oncology, Cancer Hospital and Cancer Institute, Chinese Academy of Medical Sciences and Peking Union Medical College, Beijing 100021, China. Phone: 86-10-66762694, Fax: 86-10-67715058, E-mail: zhanqimin@pumc.edu.cn.

DOK OSCC-BD

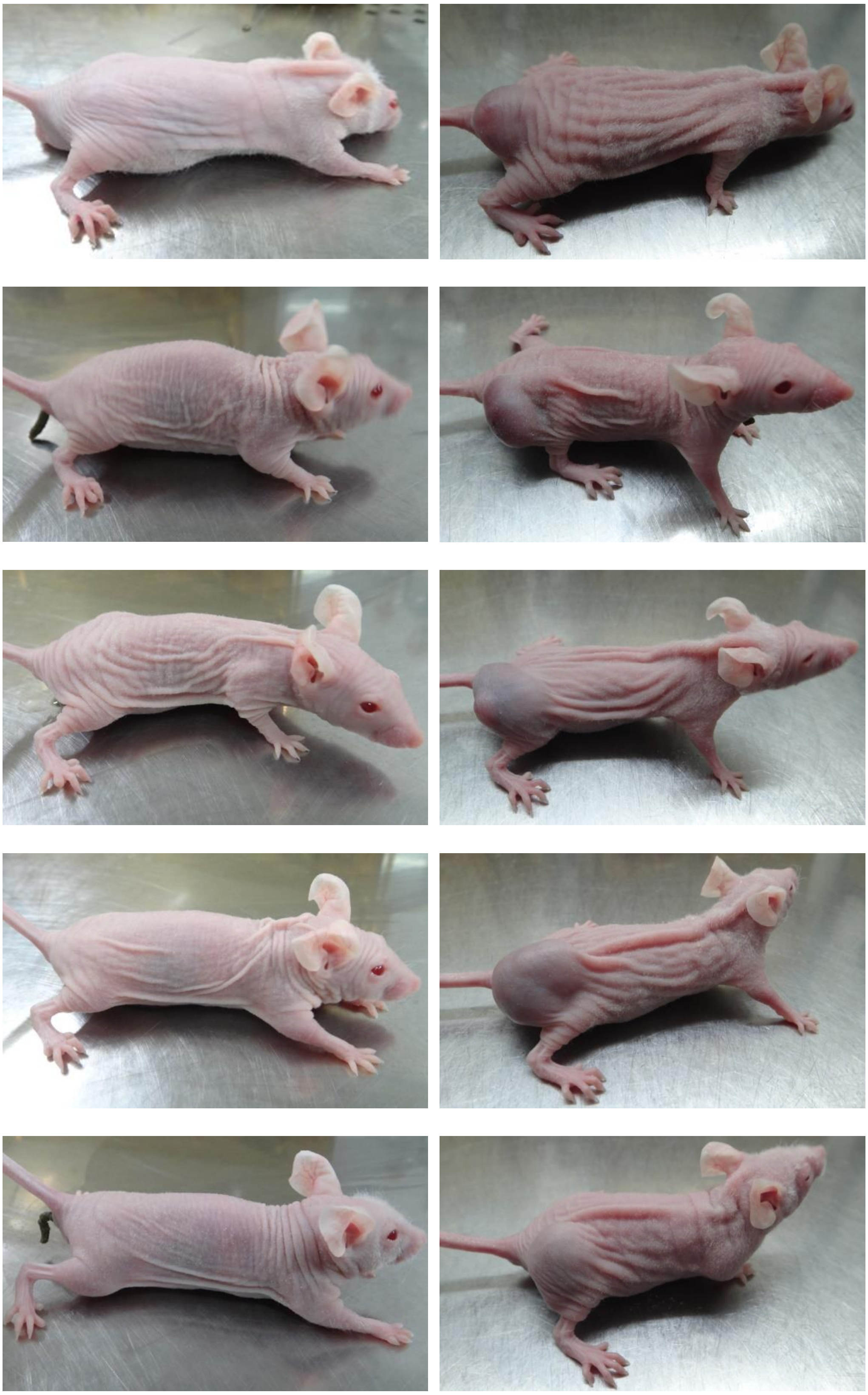

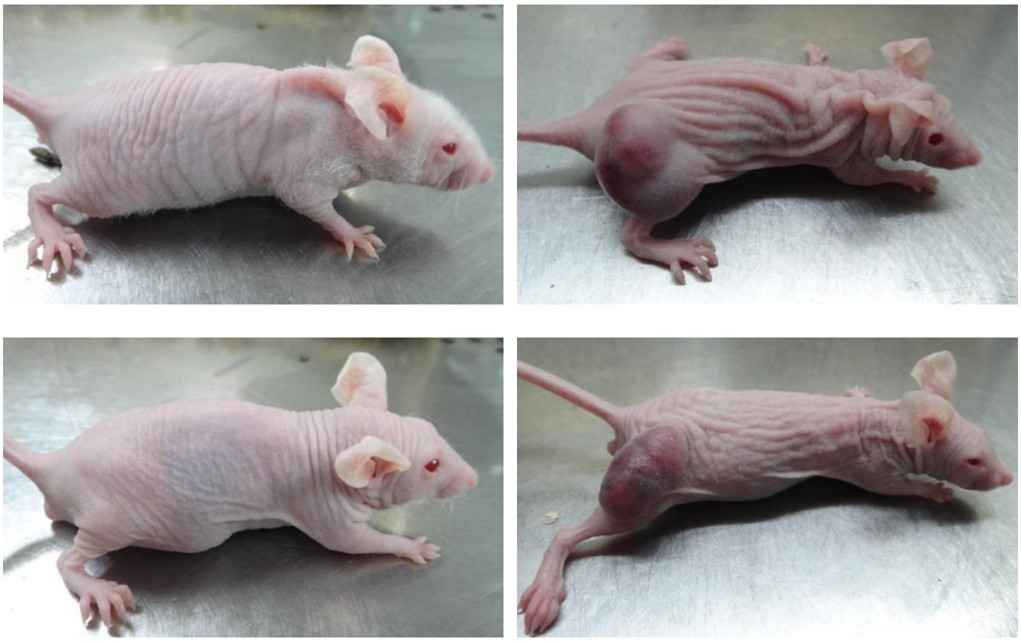

Figure S1 Two groups of nude mice (8 each) were used to analyze the tumorigenicity of DOK and OSCC-BD cells, respectively. One representative pair of nude mice was showed in Figure 5. The other seven pairs of mice were shown above. The nude mice inoculated with DOK cells are shown in the left column and no tumor was observed. The nude mice inoculated with OSCC-BD cells are shown in the right column and the rate of neoplasm formation was 100%.

Table S1. Upregulated cancer-related proteins quantified in OSCC-BD cells with RSD <8%

**Protein Name**	**Gene Symbol**	**Ratio (OSCC-BD/DOK)**	**Function Annotation***	**Biological Process***	**Biological Pathway**
Acidic leucine-rich nuclear Phosphoprotein 32 family member A	ANP32A	6.15	poly(A) RNA binding; protein binding	Transcription; Transcription regulation	
CDC21 homolog	MCM4	3.95	ATP/protein/single-stranded DNA binding; DNA helicase activity	Cell cycle; DNA replication	cell cycle control of chromosomal replication
cDNA FLJ55422, highly similar to Septin-9	SEPT9	3.89	GTP/protein binding; GTPase activity	Cell cycle; cell division	RhoA signaling; signaling by Rho family GTPases
Cytovillin	EZR	3.64	Actin filament/cell adhesion molecule/poly(A) RNA/protein binding (in epithelial cells, required for the formation of microvilli and membrane ruffles on the apical pore.)	Regulation of cell shape; Establishment of endothelial barrier	actin cytoskeleton signaling；regulation of cellular mechanics by calpain protease; RhoA signaling; signaling by Rho family GTPases
DNA helicase V	FUBP1	3.50	poly(A)RNA/protein/single-stranded DNA binding	Transcription/transcription regulation	
Tumor protein D52-like 2	TPD52L2	3.06	Poly(A) RNA/protein binding (may be a marker for breast cancer and acute lymphoblastic leukemia)	Regulation of cell proliferation	
Desmoyokin	AHNAK	2.83	Poly(A) RNA/S100 protein/protein binding; structural molecule activity conferring elasticity	Regulation of RNA splicing; regulation of voltage-gated calcium channel activity	
Activated RNA polymerase II transcriptional coactivator p15	PC4	2.71	Poly(A) RNA/protein/single-stranded DNA binding; transcription coactivator activity	Transcription/transcription regulation	
Elongation factor Tu, mitochondrial	TUFM	2.63	GTP/poly(A) RNA binding; GTPase activity	Protein biosynthesis;translational elongation	
cDNA FLJ53276, moderately similar to DNA replication licensing factor MCM2	BM28	2.39	ATP/DNA/DNA replication origin/metal ion/protein binding	Cell cycle; DNA replication	
Hsp70 /Hsp90-organizing protein	STIP1	2.35	Poly(A) RNA/protein binding	Response to stress	Endoplasmic Reticulum Stress Pathway; eNOS signaling
Elongation factor 2	eEF2	2.35	GTP/ Poly(A) RNA/protein kinase binding, GTPase activity	Protein biosynthesis	
Hemidesmosomal protein 1	PLEC1	2.09	Ankyrin/poly(A) RNA/protein binding	Apoptotic process; cell junction assembly; cellular component disassembly involved in execution phase of apoptosis	

* The function of the differential proteins was according to the Uniprot database (http://www.uniprot.org/)

Table S2. Downregulated cancer-related proteins quantified in OSCC-BD cells with RSD <8%.

**Protein Name**	**Gene Symbol**	**Ratio (OSCC-BD/DOK)**	**Function Annotation***	**Biological Process***	**Biological Pathway**
Heterogeneous nuclear ribonucleoprotein A3	HnRNPA3	0.49	nucleotide/poly(A) RNA/protein/RNA binding	RNA splicing	
Calcium pump 2	ATP2A2	0.48	Calcium-transporting ATPase activity involved in regulation of cardiac muscle cell membrane potential (catalyze the hydrolysis of ATP coupled with the translocation of calcium from the cytosol into the sarcoplasmic reticulum lumen, involved in regulation of the contraction/relaxation cycle)	Calcium transport; transport	
94 kDa glucose-regulated protein	GRP94	0.43	ATP/RNA/protein binding	ER-associated ubiquitin-dependent protein catabolic process; activation of signaling protein activity involved in unfolded protein response	
ATP synthase subunit alpha, mitochondrial	ATP5a	0.41	Mitochondrial ATP synthase;transmembrane transporter activity	ATP synthesis; transport	mitochondril dysfunction
Histone H2B	H2BFD	0.34	Core component of nucleosome	Nucleosome assembly; chromatin organization	
Vimentin	VIM	0.26	Maintain cell shape, integrity of the cytoplasm and stabilizing cytoskeletal interaction	Bergmann glial cell differentiation;host-virus interaction	14-3-3-mediated signaling；ILK Signaling; signaling by Rho family GTPases;
Isocitrate dehydrogenase [NAD] subunit alpha, mitochondrial	IDH3A	0.25	Catalyze the allosterically regulated rate-limiting step of the tricarboxylic acid cycle	Carbohydrate metabolic process; cellular metabolic process;tricarboxylic acid cycle	TCA cycle
Protein disulfide isomerase P5	PDIA6	0.20	Inhibit aggregation of misfolded proteins; play a role in platelet aggregation and activation by agonists	Apoptotic cell clearance; activation of signaling protein activity involved in unfolded protein response	

* The function of the differential proteins was according to the Uniprot database (http://www.uniprot.org/)

Table S3. Novel cancer-related proteins quantified in OSCC-BD cells with RSD <8% .

**Protein Name**	**Gene Symbol**	**Ratio (OSCC-BD/DOK)**	**Function Annotation***	**Biological Process***	**Biological Pathway**
Emerin	EDMD	3.75	Actin/beta-tubulin/protein binding	Cellular response to growth factor stimulus; regulation of canonical Wnt signaling pathway	
Inhibitor of nuclear factor kappa-B kinase-interacting protein	IKBIP	2.40	Target of p53/TP53 with pro-apoptotic function	Response to X-ray	
Clathrin light chain B	CLTB	2.25	Protein/peptide binding; structural molecule activity	Intracellular protein transport; vesicle-mediated transport	
Rab11 family-interacting protein 1	RAB11FIP1	2.14	May be involved in endocytic sorting, trafficking of proteins including integrin subunits and EGFR, and transport between the recycling endosome and the trans-Golgi network	Protein transport	
Splicing factor 9G8	SRSF7	2.09	nucleotide/poly(A) RNA/protein/zinc ion binding	RNA splicing; transport	
NHP2 non-histone chromosome protein 2-like 1 (S. cerevisiae)	NHP2L1	0.49	RNA binding	Ribosome biogenesis	
21 kDa transmembrane-trafficking protein	TMED10	0.45	Protein/syntaxin binding (act as cargo receptor at the lumenal side for incorporation of secretory cargo molecules into transport vesicles and to be involved in vesicle coat formation at the cytoplasmic side)	ER-Golgi transport; protein transport	
NADH dehydrogenase [ubiquinone] flavoprotein 2, mitochondrial	NDUFV2	0.40	Core subunit of the mitochondrial membrane respiratory chain NADH dehydrogenase	Cardiac muscle tissue development; respiratory electron transport chain	Mitochondril dysfunction
Histone H2A type 1	H2AFC	0.36	Core component of nucleosome	Nucleosome assembly	
TAR DNA-binding protein 43	TARDBP	0.34	DNA and RNA-binding protein which regulates transcription and splicing	RNA splicing; mRNA processing	
3-oxoacid-CoA transferase 1	OXCT	0.31	Key enzyme for ketone body catabolism	Cellular ketone body metabolic process	
HIRA-interacting protein 5	NFU1	0.31	Iron-sulfur cluster scaffold protein which can assemble clusters and deliver them to target proteins	Iron-sulfur cluster assembly	
Endoplasmic reticulum resident protein 70	ERP70	0.22	NADPH oxidase 1 redox signaling	Cell redox homeostasis; chaperone-mediated protein folding; glycerol ether metabolic process	

* The function of the differential proteins was according to the Uniprot database (http://www.uniprot.org/)

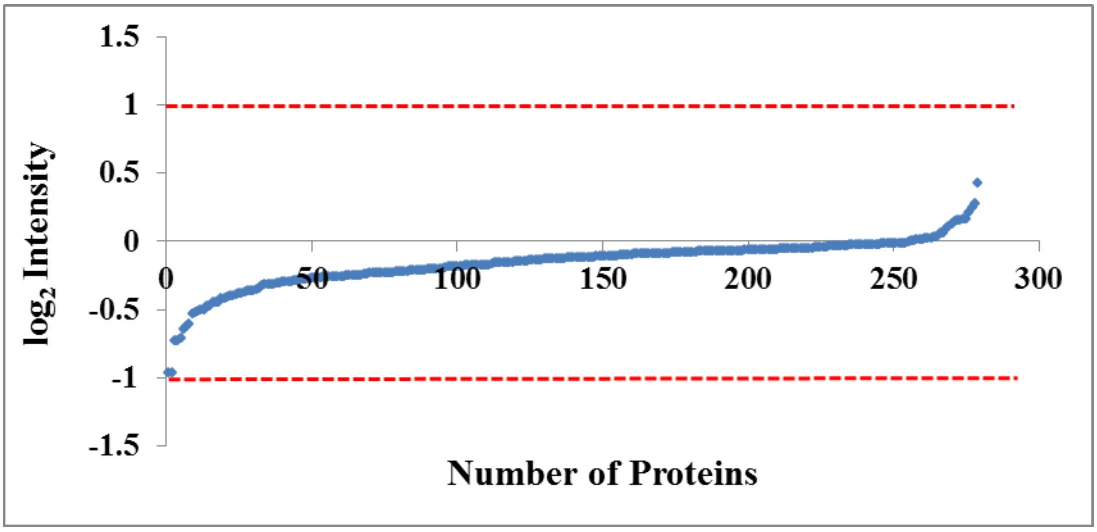

Figure S2 The log2 diagram of quantified proteins extracted from OSCC-BD cells, respectively labeled with regular and denterated formal dehyde and mixed at ratio of 1:1.

A

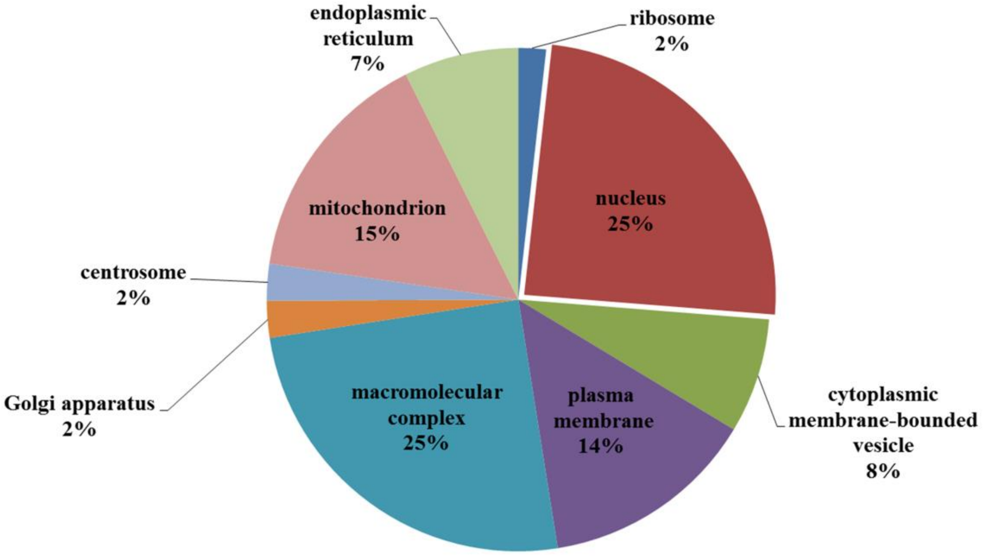

B

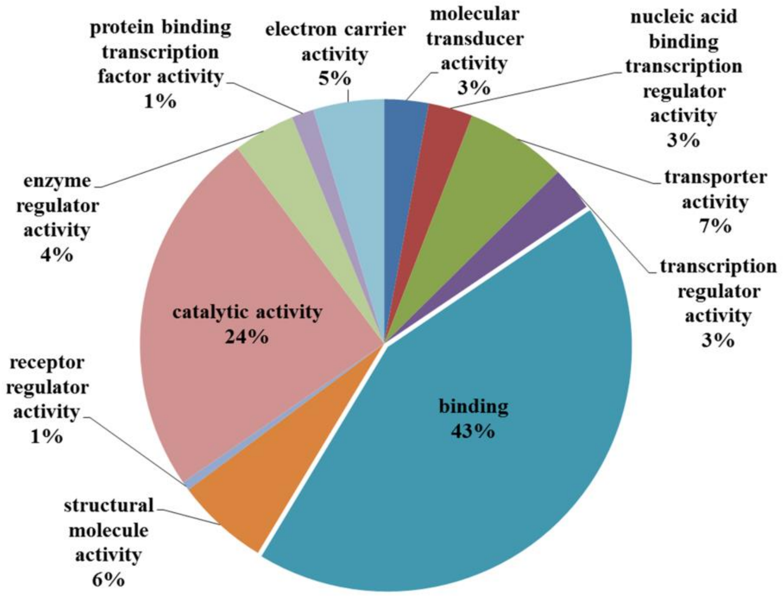

Figure S3 Distribution of cellular location and molecular function for 154 differential proteins quantified from the DOK and OSCC-BD cells. (A) Proteins mapped on the organelles of nucleus is 25% and macromolecular complex is also 25%, located in mitochondrion and plasma membrane is 15% and 14%, respectively. Other proteins were from cytoplasmic membrane-bounded vesicle, endoplasmic reticulum, Golgi apparatus, ribosome and centrosome. (B) GO molecular function annotation analysis revealed that 43% of 154 proteins were of binding function, followed by catalytic activity of 24% and transporter activity of 7%. The cellular localization and molecular functions for identified proteins based on Gene Ontology (GO) consortium were assigned with GoMiner.
